# A transition program to primary health care for new graduate nurses: a strategy towards building a sustainable primary health care nurse workforce?

**DOI:** 10.1186/s12912-014-0034-x

**Published:** 2014-12-12

**Authors:** Christopher J Gordon, Christina Aggar, Anna M Williams, Lynne Walker, Simon M Willcock, Jacqueline Bloomfield

**Affiliations:** Sydney Nursing School, University of Sydney, Sydney, NSW 2006 Australia; Australian Primary Health Care Nurses Association (APNA), Melbourne, Victoria 3205 Australia; Central Clinical School, University of Sydney, Sydney, NSW 2006 Australia

**Keywords:** Primary Health Care, Practice Nurse, Graduate Nurse, Transition, Retention, Recruitment, Workforce, Sustainability, Australia

## Abstract

**Background:**

This debate discusses the potential merits of a New Graduate Nurse Transition to Primary Health Care Program as an untested but potential nursing workforce development and sustainability strategy. Increasingly in Australia, health policy is focusing on the role of general practice and multidisciplinary teams in meeting the service needs of ageing populations in the community. Primary health care nurses who work in general practice are integral members of the multidisciplinary team – but this workforce is ageing and predicted to face increasing shortages in the future. At the same time, Australia is currently experiencing a surplus of and a corresponding lack of employment opportunities for new graduate nurses. This situation is likely to compound workforce shortages in the future. A national nursing workforce plan that addresses supply and demand issues of primary health care nurses is required. Innovative solutions are required to support and retain the current primary health care nursing workforce, whilst building a skilled and sustainable workforce for the future.

**Discussion:**

This debate article discusses the primary health care nursing workforce dilemma currently facing policy makers in Australia and presents an argument for the potential value of a New Graduate Transition to Primary Health Care Program as a workforce development and sustainability strategy. An exploration of factors that may contribute or hinder transition program for new graduates in primary health care implementation is considered.

**Summary:**

A graduate transition program to primary health care may play an important role in addressing primary health care workforce shortages in the future. There are, however, a number of factors that need to be simultaneously addressed if a skilled and sustainable workforce for the future is to be realised. The development of a transition program to primary health care should be based on a number of core principles and be subjected to both a summative and cost-effectiveness evaluation involving all key stakeholders.

## Background

In Australia and countries such as the United Kingdom (UK) and New Zealand (NZ), recent health reforms have focused on creating a responsive health system to meet the increasing challenges associated with providing cost effective, high quality and safe health care to ageing populations with growing prevalence of chronic illnesses and thus complex care needs [1,2]. There has been a policy shift away from a focus on hospital-based service provision, towards an emphasis on the future role and functions of primary health care (PHC) [3]. As a consequence, there has been an increased focus on the role of general practice [4] in meeting the health service needs and expectations of future populations. In particular, in providing community-based coordination associated with the prevention and management of chronic illnesses [1,5-8]. Concurrently, nurses working in general practice, are increasingly seen as an integral member of the PHC multidisciplinary team [9]. This is associated with increasing evidence concerning the effectiveness of their involvement in chronic illness prevention and management [4] and the role they continue to play in maintaining the capacity of PHC services, particularly in rural areas [10]. The ongoing development of the PHC nursing workforce in Australia is considered important in safeguarding the development of a skilled and sustainable PHC workforce to meet future needs of the population [1].

The future sustainability of the PHC nursing workforce is, however, threatened by an ageing workforce and workforce shortages [11-13]. Innovative solutions that aim to address PHC nursing supply and demand issues are urgently required. This debate article describes the PHC nursing workforce dilemma facing policy makers in Australia today and advocates for a New Graduate Nurse Transition to Primary Health Care Program, as a potential PHC nursing workforce recruitment and retention strategy. It is postulated that a New Graduate Nurse Transition to Primary Health Care Program will contribute to the development of a skilled and sustainable PHC nursing workforce for the future.

## Discussion

### Primary health care nurses working in general practice in Australia and the workforce dilemma

In Australia, a PHC nurse working in general practice is defined as a degree-qualified registered or certificate-qualified enrolled nurse [5]. These PHC nurses are engaged in the provision of a range of initiatives including health promotion and prevention (e.g. immunisations, health checks, health education), clinical care (e.g. emergency care, triage, specific illness education, wound management) and care coordination (case management, preparation of chronic illness and multidisciplinary care plans and patient referral) [7,10].

Over the past 15 years, considerable concurrent investment has been made by the Australian government and key medical, nursing and professional support organisations, to support the development of PHC nursing. Initiatives have focused on defining and developing the role of PHC nurses working in general practice. This has included the development of competency standards [14], the introduction of tertiary-based postgraduate education, the implementation of incentives for nurses to work in general practice (e.g. scholarship schemes) [15], and for general practices to employ nurses (e.g. Nursing in General Practice Program (NiGP) and the Practice Nurse Incentive Program (PNIP) [7]. Despite the sustained investment, and in contrast to countries such as the UK and NZ, PHC nursing in Australia has yet to evolve into a recognised speciality [4] and the work of nurses in general practice remains largely task focused [10]. Australian government-funded initiatives such as the Strengthening Medicare Package and the subsequent additional Medicare Benefit Schedule items [16], have been criticised for promoting a task-oriented role for nurses working in general practice [9]. Today, the PHC nursing profession in Australia continues to grapple with a number of professional issues including the lack of: a comprehensive educational framework for nurses working in general practice [15,17], a defined career framework [5] and an agreement on models of practice and the role of PHC nurses as part of a multi-disciplinary team [10]. In addition, PHC nurses report limited access to or uptake of post-graduate education in PHC opportunities, despite the introduction of Federal Government scholarship initiatives [5]. Ultimately, these issues continue to negatively influence the recruitment and retention of PHC nurses [17]. To date, strategies aimed at increasing the numbers of PHC nurses working in general practice have focused on retaining the existing workforce and supporting the transitioning of experienced registered nurses from the acute care hospital sector to the PHC sector. Although these strategies have had some success, resulting in an increase in the total number of nurses employed in general practice, from approximately 8914 nurses in 2009, to 10,693 nurses in 2012 [7], a shortfall in the overall number of PHC nurses remains [18]. It is predicted that by 2025, Australia will face an overall nursing workforce deficit of approximately 110,000 nurses [11,12] and the impact on general practice will be pronounced.

The retention of nurses in the workforce is considered the most significant factor in reducing predicted nursing shortages [19]. Retaining nurses currently working in general practice is important in maintaining the current workforce and a level of leadership to educate and support nurses entering the profession in the future. This approach in isolation however, will not produce workforce stability in the long-term. Currently PHC nurses are an ageing workforce, where the majority of nurses working in general practice are aged over 40 years (81.3%), with an alarming 40.8% of these nurses, aged between 50-59 years [7]. Therefore, novel solutions to engage nurses to work in general practice is required to both increase workforce numbers and create sustainability in PHC nursing.

A national nursing workforce plan that adequately addresses issues that are impacting on the supply and demand of PHC nurses and builds the capacity of the workforce to meet the future needs of the population is required [17]. Innovative solutions must be sought, developed and tested that will support and retain the current PHC nursing workforce, whilst building a skilled and sustainable PHC nursing workforce for the future. One proposed innovation is the recruitment of new graduate nurses to work in general practice, immediately following completion of a pre-registration degree and attainment of nursing registration.

In Australia, since the introduction of university-based education for nurses in the 1980s, newly qualified registered nurses typically enter the workforce through a transition to practice program. At present, there is a surplus of new graduate nurses resulting in limited placements [20]. Therefore, the addition of a transition program for new graduate nurses into PHC will increase employment opportunities, and may inevitably increase current and predicted nursing shortages in the future [20-23].

Transition to practice programs acknowledge that new nursing graduates require additional education preparation and consolidation of skills to support the evolution from student nurse to a practising professional [24,25]. Similarly, new graduate nurses specifically recruited to work in a general practice setting, would also require the support provided by a transition program - to access specific education and develop the necessary skills to work effectively in this field. However, there is a paucity of information on transition programs for graduate nurses entering general practice settings. Therefore, this innovation would require the development and evaluation of a transition to practice program specifically for graduate nurses entering the nursing profession in a general practice setting.

A number of questions would need to be explored in the development of a New Graduate Nurse Transition to Primary Health Care Program:

1. To what extent do existing transition to practice program models inform the development and implementation of a New Graduate Nurse Transition to Primary Health Care Program?
2. What is known about the efficacy, effectiveness and cost-effectiveness of transition to practice programs?
3. How should a New Graduate Nurse Transition to Primary Health Care Program be structured and what are the key components to be included?
4. What factors will contribute to or hinder the success of a New Graduate Nurse Transition to Primary Health Care Program?

### Existing transition to practice models to inform program design, implementation and evaluation

Internationally there are various models of transition to practice programs that are more frequently designed for and implemented in acute care settings [26]. The programs generally aim to: (1) develop new graduates into proficient and confident registered nurses; (2) support the evolution of the student to a practising professional; and (3) maintain the graduates commitment to a career in nursing [25].

Some countries have developed a national standardised transition to practice program such as the Flying Start Program in Scotland [24] and the United States National Council of Nursing’s Regulatory Model of Transitioning New Nurse to Practice [27]. New Zealand has an entry to practice program that is implemented in different sites across District Health Boards [28]. In Australia, there is a variation in the number and type of programs that exist between states, although most are implemented locally within health services attached to state based health districts or networks. Core components of transition programs typically include a period of orientation, with supernumerary time and study days, combined with mentoring or preceptorship [25]. Some programs have included elements such as “academic credit-status” points, that when accrued can be used towards university based postgraduate studies [28]. This has been undertaken to create incentives for the new graduate nurse to undertake postgraduate speciality studies in the future.

Preceptorship, where a senior nurse clinician provides ongoing clinical teaching and instruction, with the aim of expanding the graduate nurse competence and confidence [29], stands out as an important component to include in a transition program. Supernumerary time is also important to allow the new graduate to grow into their role without workload pressures [26]. Preceptors require training to develop the necessary facilitation and relational skills [29] with training covering principles of adult learning, learning styles, an overview of the transition to practice theory and conflict resolution strategies [26].

Existing transition to practice programs report differences related to education programs program structure, content, funding models and length of duration [25,30]. Education strategies used in transition programs have included course work and classroom sessions (covering clinical and professional development topics), patient simulation, journal assignments, professional portfolios and “blended learning models” that utilise e-learning activities [31]. However, there is a lack of detail in the literature concerning the extent to which the different education strategies have been used and their impact [26].There are contrasting opinions regarding the number of different clinical areas that graduates should be exposed to in a transition program, what is considered the optimal length of new graduate programs (ranging from 12 weeks to 12 months) and when transition programs are most beneficial [31].

There is a general lack of information in the published literature regarding models of funding for transition programs. In Scotland, a transition to practice program was funded by the Executive Health Department to the National Health Service Boards to support the implementation of The Flying Start Program nationally [24]. In NZ, new entry to practice programs are funded collaboratively between the ‘central government’ and District Health Boards [29]. In Australia, public health service based transition programs are funded by State and Territory governments with significant variations reported in the level of funding received by programs between each State and Territory [25].

### The efficacy, effectiveness and cost-effectiveness of transition to practice programs

There is minimal published evidence concerning the efficacy [30] and at times, conflicting evidence in regards to the effectiveness of different components of the programs [2] or the different program types [32]. There is a need for further research and evaluation concerning the efficacy, effectiveness and cost-effectiveness of the programs [30] and investigation of the potential barriers faced by transition to practice programs.

Nevertheless, a number of studies have reported positive outcomes associated with transition to practice programs, including increased confidence, competence and satisfaction of new graduates with their employment [29]. Program components such as mentorship and preceptorship have been associated with a reduction in “transition shock” [25], role socialisation [29] and retention of new graduates [26,29]. In Australia, new graduate nurses transitioning into the acute care sector have been found to be receptive to, and value the support of transition to practice programs [25,33]. In addition, new graduates report a “sense of belonging” to the setting in which they completed their transition program [34] and have expectations that their employers will provide support in their work environment to enable them to progress from student to professional nurse [35]. Although in Australia there have not been transition to practice programs for nurses into PHC, nurses working in general practice have been found to be enthusiastic towards undertaking a mentoring role with student nurses on clinical placements [4,18]. Nurses working in rural areas have identified positive outcomes associated with undertaking a preceptor role, including a sense of personal achievement and opportunities for development [36].

### Proposed structure and core components of a New Graduate Nurse Transition to Primary Health Care Program in Australia

A number of key principles should govern the overall design, implementation and evaluation of a graduate nurse transition to Primary Health Care Program (see section: Governing principles associated with the design, implementation and evaluation of a Graduate Nurse Transition program to Primary Health Care).

### Governing principles associated with the design, implementation and evaluation of a Graduate Nurse Transition program to Primary Health Care

#### Program design

1. The inception and design of the transition program should involve all relevant key stakeholders including representatives from national and state health departments, primary health care organisations, general practice, medical and nursing professional organisations and other interested parties;
2. A national needs assessment engaging all key stakeholders should inform the design, implementation and evaluation of the program;
3. The program should be designed as a national, structured program to promote consistency in the education and support provided for Practice Nurses;
4. The design of key program elements should incorporate existing professional standards and frameworks such as the competency standards for nurses working in general practice;
5. The educational component of the program should focus on the development of entry level skills as a primary health care nurse working in general practice and adopt a preceptorship model to ensure adequate supervision and support of the graduate nurses* and support and training for the participating practice nurses;

#### Program implementation

6. A pilot evaluation to test the feasibility, acceptability and scalability of the program should be conducted initially;
7. Government funding to support a Graduate Nurse Transition program to Primary Health Care would align with current financial methods to support workforce development and training in general practice;
8. Primary health care organisations should govern and support the implementation of the national program at local levels;
9. A competitive but transparent process should be developed to support the recruitment of new graduate nurses to the program;
10. Energy should be spent on investigating financial and other incentives that could be used to promote engagement with the program by key stakeholders, in particular, general practitioners, existing primary health care nurses working in general practice and student nurses in their final year of pre-registration studies;

#### Evaluation

11. Current and future roles of nurses and thus changes in their education, skill development, supervision and support needs, should be captured in quality assurance mechanisms associated with ongoing program development and evaluation;
12. Key Performance Indicators and outcomes of interest associated with the Program should be developed collaboratively with involvement from all key stakeholders and have a specific focus on the contribution of the transition program in developing a skilled and sustainable practice nursing workforce; and
13. A comprehensive evaluation that investigates the efficacy, effectiveness and cost-effectiveness of the program should be undertaken, with the results widely disseminated.

*A Mentor is concerned with the development of a more long term relationship with a nurse that focuses on their professional development and growth. Whereas, the Preceptor engages in more short-term clinical teaching and instruction with a focus on orientation and role socialisation with the aim of building the nurse competence and confidence [29].

Development of the program in partnership with key stakeholders will be paramount in ensuring sustainability. An initial priority in program inception will be to undertake a national needs assessment of staff within general practices and final year pre-registration nurses to provide critical information related to attitudes, knowledge, skills and support needs to inform program design and delivery.

The New Graduate Nurse Transition to Primary Health Care Program should be designed as a national structured program to ensure consistency in the training, ongoing education and support provided to participating new graduates and existing PHC nurses and to align with the current structure and function of general practice in Australia [24]. The program should however, be designed with inbuilt flexibility to support delivery in different States and Territories and according to differences in the organisation and environment of general practices. Primary Health Care organisations at regional levels might be ideally suited to govern and coordinate the implementation of such a program within general practices.

A New Graduate Transition to Primary Health Care Program is likely to contribute to the development of nursing in general practice as a speciality area and a career framework for PHC nurses. One possible outcome of the program is the recognition of a formal tier structure of nurses working in general practice. This would include the recognition of the higher level of experience of PHC nurses who have undertaken training and performed the role of a preceptor associated with a transition program.

In regards to workforce development and training (excluding rural General Practice incentives), general practices receive financial assistance from the Australian Government through the Practice Incentive Program (PIP) which is associated with medical student teaching and the PNIP which is associated with the employment of nurses in general practice. Federal government funding to support a New Graduate Nurse Transition to Primary Health Care Program would align with current financial methods to support workforce development and training in general practice. Possibilities to consider include the remuneration of general practices for the placement of pre-registration nursing students, similar to the PIP supporting medical student placements to expose students to general practice during their training. In addition, postgraduate training support models for general practice such as the Prevocational General Practice Placement (PGPP) or the Australian General Practice Training (AGPT) programs could be expanded or modified to include nurses. This might be justified on the provision of financial assistance to allow practices to offset the cost and time spent by the PHC nurse preceptors and the potential supernumerary time of the new nurse graduates [4]. It is envisaged that funding of transition programs could be built on existing initiatives such as the PNIP to support practices to employ the new graduate nurses. Financial support for PHC organisations to coordinate and implement the program including training and support of PHC nurses to engage as preceptors would also be required.

An integral component of a transition to Primary Health Care Program will be comprehensive formative and summative evaluations, including cost-effectiveness studies. To support evaluations, key performance indicators and targeted outcome measures should be defined and evaluated. These indicators at a minimum should focus on the level of competency and confidence obtained by the new graduates and their intention to stay in the nursing workforce and in the field of general practice in the future. Evaluations should also include the impact of engagement with the program on the existing PHC nurse workforce and the service delivery of general practices.

### Factors contributing to or hindering the success of a transition programs

Support from all key stakeholders including financial support from the Australian government will be essential for the development and evaluation of a New Graduate Transition to Primary Health Care Program.

The capacity of general practice to be able to support the employment of new nurse graduates, in addition to current nursing staff, will be important to ascertain in the initial needs assessment. Physical size constraints of practices have been reported to impact on the number of nurses employed in general practices and on the PHC nurses’ role [4]. Investigating the optimal size and work environments of general practices, will be important in developing recruitment protocols as part of a program implementation plan. Evaluating the impact of the program on the workload of the PHC nurse preceptor will also be required.

The literature highlights the importance of the quality of the preceptorship received by new graduates in achieving their retention as an outcome. The confidence of new graduates may be affected negatively by inexperienced or poorly motivated preceptors [29]. It will be important that a selection criteria is developed for engaging experienced and motivated PHC nurses into the role of a preceptor and that they are in addition provided with sufficient education and training in transition theory and the needs of new graduate nurses, as well as being provided with support and feedback by the coordinators of the program. Investigating other incentives such as business case studies to encourage general practices to collaboratively engage and support the program will be important. Linking the transition to practice program to obtaining credit points associated with gaining a formal post-graduate tertiary qualification in PHC, may increase the interest of pre-registration students in taking part in the program and partially remove barriers associated with completion of post-graduate studies [23]. The success of a New Graduate Transition to Primary Health Care Program, to some extent, will be dependent on the profession concurrently, reaching an agreement on and developing an educational framework for PHC nurses, preferred models of practice and a defined career pathway. In addition, securing funding to support ongoing program implementation and evaluation will be paramount. Establishment of these elements will be important in being able to promote PHC nursing within general practice settings as a viable career option for nurses. This may attract the interest and commitment of new graduate nurses to participate in a New Graduate Nurse Transition to Primary Health Care Program and build a career in PHC in the future.

## Summary

Australia, like other developed countries has an ageing population, with increasingly complex health needs due to the increased prevalence of chronic illness and multi-morbidity and a corresponding current and predicted future health workforce shortages. A transition program to primary health care for new graduate nurses, similar to the current acute care hospital-based sector, may be a viable option to address these deficiencies. A program would provide a formally structured pathway for new graduate nurses to enter PHC settings underpinned with a supportive learning environment in the first year, which has been shown to be crucial to development of proficient, confident registered nurses. Issues of transition logistics would need to be considered, however, it is proposed that this initiative would address several complex workforce issues relevant to general practice and create a viable workforce model for the future.

## Abbreviations

UK: United Kingdom
NZ: New Zealand
PHC: Primary Health Care
PHCN: Primary Health Care Nurse (Nursing)
NiGP: Nursing in General Practice Program
PNIP: Practice Nurse Incentive Program
PIP: Practice Incentive Payment
PGPP: Prevocational General Practice Placement
AGPT: Australian General Practice Training
